# Notch/CXCR4 Partnership in Acute Lymphoblastic Leukemia Progression

**DOI:** 10.1155/2019/5601396

**Published:** 2019-06-27

**Authors:** Georgia Tsaouli, Elisabetta Ferretti, Diana Bellavia, Alessandra Vacca, Maria Pia Felli

**Affiliations:** ^1^Department of Molecular Medicine, Sapienza University of Rome, Viale Regina Elena 291, 00161 Roma, Italy; ^2^Department of Experimental Medicine, Sapienza University of Rome, Viale Regina Elena 324, 00161 Roma, Italy

## Abstract

Acute lymphoblastic leukemia (ALL) is the most common cancer among children. Recent advances in chemotherapy have made ALL a curable hematological malignancy. In children, there is 25% chance of disease relapse, typically in the central nervous system. While in adults, there is a higher chance of relapse. ALL may affect B-cell or T-cell lineages. Different genetic alterations characterize the two ALL forms. Deregulated Notch, either Notch1 or Notch3, and CXCR4 receptor signaling are involved in ALL disease development and progression. By analyzing their relevant roles in the pathogenesis of the two ALL forms, new molecular mechanisms able to modulate cancer cell invasion may be visualized. Notably, the partnership between Notch and CXCR4 may have considerable implications in understanding the complexity of T- and B-ALL. These two receptor pathways intersect other critical signals in the proliferative, differentiation, and metabolic programs of lymphocyte transformation. Also, the identification of the crosstalks in leukemia-stroma interaction within the tumor microenvironment may unveil new targetable mechanisms in disease relapse. Further studies are required to identify new challenges and opportunities to develop more selective and safer therapeutic strategies in ALL progression, possibly contributing to improve conventional hematological cancer therapy.

## 1. Introduction

The most common childhood malignancy, acute lymphoblastic leukemia (ALL), originates from malignant transformation of B- (80-85%) or T-cell (20-25%) precursors. Pediatric ALL is highly responsive to chemotherapy; however, 15-20% of children still experience disease relapse [1]. In contrast, approximately 50% of adults are affected by ALL relapse after treatment [1]. Leukemic infiltration of the liver, spleen, lymph nodes, and mediastinum is common at diagnosis. Extramedullary infiltration by leukemic cells also involves the central nervous system (CNS) and testicles thus requiring a specific therapy [2]. In recent decades, clinicians have seen a significant improvement in event-free survival rates [3], but an accurate diagnostic process is needed to support an optimal risk-oriented therapy and thus to increase the cure rate. Immunophenotyping of ALL represents the diagnostic gold standard for the identification of the cell lineage and the specific subset and represents also a useful tool to detect and to monitor minimal residual disease. In B-lineage, the most important markers are CD19, CD20, CD22, CD24, and CD79a, while CD1a, CD2, CD3 (membrane and cytoplasm), CD4, CD5, CD7, and CD8 for the T-lineage ALL [4]. The unequivocal diagnosis of T-ALL requires the detection of surface/cytoplasmic CD3 [4]. Genomic heterogeneity characterizes different molecular subtypes of ALL, and new entities have been recently identified [5]. MicroRNA (miRNA) expression profiling, by identifying possible biomarkers, may be useful in differential diagnosis [6]. Infiltration of the CNS is a common risk, but bone marrow (BM) assessment is the first step in the diagnostic pathway [7]. More than 60% of T-ALL patients display gain-of-function mutations in Notch1, resulting in a ligand-independent signaling [8]. The study of Bernasconi et al. [9] evidenced the presence of Notch3-activating mutations in human T-ALL. Recently, Notch1 and Notch2 have also been implicated in subsets of mature B-cell malignancies [10, 11]. Therefore, Notch signaling is a common denominator in ALL disease.

The chemokine ligand/receptor system has been implicated in the regulation of organ-specific infiltration during metastasis. CXCR4 (CD184) is the chemokine receptor specific for CXCL12, also termed stromal cell-derived factor-1 (SDF-1/CXCL12), which is released by the stromal cells resident in the thymus and in the BM. Genetic disruption of either SDF-1 or CXCR4 in mice is lethal [12]. Under physiological conditions, the SDF-1/CXCR4 signaling is critical not only to the retention of hematopoietic cells (such as CD34+ stem cells and B-cell precursors) in the BM but also for tissue dissemination and localization of T-cells and other mature hematopoietic cells [13]. The overexpression of CXCR4 on the surface of solid and hematological tumors is strictly related to disease progression [14]. Interestingly, oncogenic Notch activation enhances CXCR4 cell surface expression in T-ALL [15–17].

In this review, we discuss recent insights into the relevant roles played by Notch and CXCR4 in the pathogenesis of B- and T-ALL development and progression. First, we review the deregulated Notch and CXCR4 signaling pathways comparatively between B- and T-ALL. Then, we survey recent efforts aimed at unraveling the mechanisms involved in ALL progression and the role played by the partnership of Notch and CXCR4 receptors.

## 2. The Notch Pathway: An Overview

Mammalian Notch receptors comprise four paralogues (Notch1-4). Notch signaling is a cell-to-cell communication pathway triggered by five cognate ligands, Delta-like (DLL1/3/4) and Jagged families (Jagged1/2), expressed by signal-sending cells [18]. Notch receptors are expressed as transmembrane heterodimers after furin-like cleavage (S1) that occurs before their transit to the cell surface. Upon ligand binding, a physical force exposes a proximal region of the Notch extracellular domain to cleavage by ADAM10 metalloproteases (S2) and then *γ*-secretase (S3) [19]. The generated active intracellular domain of Notch (NICD) translocates into the nucleus where it interacts with the transcription factor RBPJ (also known as RBP-Jk or CSL) and a mastermind-like family (MAML) transcriptional coactivator. The NICD-RBPJ-MAML complex activates target gene transcription in cooperation with other transcription factors and epigenetic regulators.

NICD has a short half-life due to rapid proteosomal degradation regulated mainly by the C-terminal PEST domain [region rich in the amino acids proline (P), glutamic acid (E), serine (S), and threonine (T)], which serves to recruit the FBXW7 protein, an E3 ubiquitin ligase implicated in NICD turnover [20]. In fact, ankyrin domain (ANK) and PEST sequence regulate protein stability and degradation. Only Notch1 and Notch2 include a transactivation domain (TAD) between ANK and PEST [21].

Notch has a major effect on T-cell lineage commitment. Notch1 [22] and Notch3 [23] regulate different intrathymic T-cell developmental stages, whereas Notch2 plays a key role in the progression of transitional B-cells to marginal zone B-cells [24]. In mature B-cells, Notch1 expression is increased markedly either by activation of B-cell receptor signaling or by lipopolysaccharide (LPS) and plays a role in terminal differentiation of B-cells [25, 26].

## 3. The Oncogenic Activity of Notch in B-Cell Malignancies

Oncogenic Notch activation is not a characteristic of B-ALL (Table 1), a precursor B-cell neoplasm, but mutations of Notch genes have been identified in neoplasms of mature B-cells [8]. Instead, Notch1-activating mutations are present in 4-13% of B-cell chronic lymphocytic leukemia (B-CLL) cases [27], but nonmutational Notch1 activation has also been reported in B-CLL [28]. Genetic alterations in Notch genes, mainly in Notch1 and Notch2, occur in other B-cell malignancies such as CLL, splenic marginal zone lymphoma, mantle cell lymphoma diffuse large B-cell lymphoma (DLBCL) and, rarely, follicular lymphoma [8, 29]. Moreover, Notch in mature B-cell and therapy-resistant B-cell malignancies (such as Hodgkin lymphoma, multiple myeloma and mixed-lineage leukemia translocated cell lines) is a potent inducer of growth arrest and apoptosis [30], while in B-CLL cells [31], it promotes survival and apoptosis resistance.

Gain-of-function mutations of Notch1 and Notch2 are localized essentially in the PEST domain, but loss-of-function mutations of negative regulators of the Notch pathway, such as Deltex1 (DTX1) and SPEN (spen family transcriptional repressor), have been also described in aggressive large B-cell lymphoma; these mutations still need to be molecularly dissected [27, 32, 33]. Furthermore, to date, the role of Notch ligands inducing Notch activation in B-cell lymphomas is still obscure, although it has been demonstrated that B-cell lymphoma cells express Notch ligands [34]. The role played by Notch ligand presenting cells within the tumor microenvironment still needs to be studied. Notch synergizes with B-cell receptor (BCR) signaling by enhancing B-cell activation, thus suggesting a possible cooperation between Notch and BCR receptors with microenvironment inputs in B-cell lymphoma [8]. In DLBCL, a novel immunomodulatory function of B-cells has been proposed where aberrantly activated Notch1 signaling drives T-cell immunity towards Treg and Th2 cell-dominant responses are mediated principally by IL-33. Thus, in DLBCL, Notch-activating mutations promote immune evasion of mature B-cell neoplasms [34].

B-ALL is a more common and heterogeneous disease than T-ALL; however, there is no clear evidence of Notch mutations in B-cell context (Table 1). Somatic mutations in B-ALL include genes associated to differentiation and development (18%; e.g., Pax5 and IKAROS), Ras-signaling, Jak/STAT signaling, cell cycle regulation, and tumor suppression [35]. Nevertheless, a possible epigenetic regulation of Notch genes in B-ALL has been hypothesized. Indeed, Notch receptor and their target genes are frequently hypermethylated, thus leading to Notch-Hes1 axis inactivation [36]. Moreover, Notch3 and Hes5 were found preferentially hypermethylated in B-lineage lymphoblastic cell line and primary B-ALL [37]. Therefore, demethylating and deacetylating agents can relieve the epigenetic suppression of Notch genes possibly with the aim at reversing B-cell leukemia-uncontrolled proliferation and apoptosis resistance. Interestingly, mutation of FBXW7, an ubiquitin protein ligase, which regulates levels of NOTCH, has been found in four of 118 B-ALL and quite frequently in T-ALL patients. All mutations affected the FBXW7 target-interacting domain. These observations suggest that disruption of FBXW7 is involved in several forms of lymphocytic leukemia and it is not exclusive of T-ALL [38].

It has been observed that Notch ligands, expressed by follicular dendritic cells, protect B-cells from apoptosis in germinal centers [39]. High expression of Notch ligands (Jagged2, DLL3/4) and receptors (Notch1/3/4) has been reported in a subset of B-ALL patients. In this context, BM stromal cells contribute to the survival of B-ALL cells by activating Notch signaling. This evidence may highlight the importance of intercellular signaling between B-ALL cells and their microenvironment [40]. Recently, Notch3 and Notch4 receptors have been implicated in supporting survival of primary B-ALL, also suggesting a potential role in drug response. In fact, the authors demonstrated that Notch inhibition, by gamma-secretase inhibitors XII or IX and/or anti-Notch4 blocking antibody, could sensitize B-ALL cells to apoptosis induced by cytarabine, dexamethasone, or doxorubicin [41]. Furthermore, a recurrent translocation *t*(14; 19) (q32;p13) in B-ALL has been associated to deregulation of Notch3 and/or ABHD9 genes [42]. To gain more insights into the role of Notch, a new B-ALL cell line with constitutional Notch signaling defect has been derived from the BM of a patient affected by both B-ALL and Alagille syndrome. Multiple aberrations in components of Notch signaling, including Notch1 and Notch3, were described [43]. Due to limited information, the crosstalk between active Notch signaling and other oncogenic pathways in B-ALL still needs to be elucidated.

## 4. To Notch Up T-ALL Disease

Notch is frequently found deregulated in T-ALL. For that reason, mutations directly to a specific Notch gene (Notch1, Notch2, or Notch3) (Table 1) or in genes codifying for partners of the Notch pathway (FBXW7, Notch ligands, or MAML1) as well as epigenetic modulations of Notch in T-ALL are examined in this paragraph. In addition, we will discuss the impact of other activated signaling pathways on Notch and how different gene products can modulate the latency of the disease.

In contrast to B-ALL, activating mutations of Notch1 are common in human T-cell leukemia [8, 37]. This mutated receptor represents the most frequent oncogene across all subtypes of T-ALL [44]. Most mutations of Notch occur in two areas, either in the negative regulatory region (NRR) or in the C-terminal PEST domain. Missense mutations, deletion, or short insertions usually target the extracellular NRR, which contains the heterodimerization domain (HD). These alterations lead to receptor destabilization and proteolytic activation in the absence of the ligand. On the other hand, nonsense or frameshift events that insert premature stop codons occur in C-terminal PEST domain of Notch, resulting in an increased half-life of the receptor. Mutations in the Notch2 gene have been found specifically in adult T-ALL cases [45]. Although Notch3 is rarely mutated [9], it is very frequently hyperexpressed in the majority of T-ALL cases [37, 46] and a subset of human T-ALL depending on Notch3 mutations has been recently described [9].

Interesting studies revealed that inactivating mutations in FBXW7 gene decrease ICN degradation and together with Notch1-activating mutations are found in 8-30% of T-ALL patients [47]. The simultaneous presence of these two mutations results in increasing the half-life of Notch1-receptor and thus in reinforcing Notch signaling in malignant cells. Moreover, also the aberrant expression of the Notch ligand, DLL4 or Jagged1, may contribute to Notch-driven leukemia [48, 49].

The Notch pathway can also be epigenetically regulated, underlying an alternative way for its activation. In fact, Notch (Notch3) and its signaling target (HES5) are poorly methylated or even unmethylated in T-ALL cells [36]. This would suggest that while demethylating and/or deacetylating agents can counteract B-cell leukemia, they would be ineffective in T-ALL. Recently, it has been demonstrated that the inactivation of an epigenetic regulator, histone deacetylase 6 (HDAC6), is followed by increased lysosomal localization of Notch3, which correlates with reduced Notch signaling strength [50]. Moreover, activated Notch1- or Notch3-ICD can bind to the Notch3 gene locus and by recruiting H3K27 modifiers (JMJD3, p300) can sustain the expression of Notch3 and of its targets such as DTX1 and c-Myc [51]. Therefore, JMJD3 and p300 can be considered general coactivators of Notch1 and Notch3 signaling in T-ALL.

A crucial coactivator of all four Notch receptors is MAML1, which through the NICD-RBPJ-MAML complex regulates most of the Notch function in cell proliferation, survival, and differentiation. Recently, it has been demonstrated that MAML1 knocking down inhibits proliferation and induces apoptosis of T-ALL cells [52].

Intersection of the Notch pathway with other signaling pathways, including NF-*κ*B, may enhance the oncogenic activity of Notch but may also give a new way to tackle Notch hyperactivation. Downstream targets of Notch signaling can be considered not only the direct ones, like MYC, HES1, HES5, or IL7R, but also the indirect ones, such as PI3K/AKT/mTOR signaling pathway components [8] (and references therein). Deregulation of the last one is associated to poor prognosis and limited response to therapy in T-ALL, and it may be also the target of the combined therapy with Notch inhibitors as demonstrated in *in vivo* assay [53].

Both hyperactive Notch1 and Notch3 can enhance CXCR4 cell surface expression in thymus-derived and in BM-derived T-cells [14–16]. The intersection of these two pathways plays also an important role in T-ALL. In Notch1-induced T-ALL cells, CXCR4 silencing inhibited the expansion of leukemic cells due to increased cell death and also altered cell cycle progression [15, 16]. In Notch3-induced T-ALL cells, enhanced CXCR4 surface expression is correlated to a high proliferative rate and percentage of Ki67-positive thymocytes [17, 54]. These results suggest that the cooperation of either Notch1 or Notch3 with CXCR4 converges in increasing the proliferative programs in leukemia cells, making them more competent to infiltrate other immune districts.

Developmental pathways are often disrupted in leukemia. Several reports have demonstrated that the Hedgehog (HH) signaling is implicated in T-ALL, while only few evidences have been reported in B-ALL, where the HH pathway specifically targets the self-renewal of B-ALL cells [55]. The role of HH in T-ALL is controversial. A study showed that HH signaling is dispensable for T-ALL development in a Notch1-ICN-dependent mouse model [56], while other studies demonstrated sensitivity of T-ALL cell lines to HH inhibitors [57, 58]. Activating mutations of HH signaling partners have been documented in T-ALL [59], but only a subset (20%) of T-ALL cases shows the activation of the HH pathway by displaying high expression of HH ligands and of the downstream Gli transcription factor [60]. HH ligands bind to thymic epithelial cells and induce the expression of T-ALL-promoting proteins such as the Notch ligand DLL4, IL-7, and VEGF. Moreover, HH stimulation in two T-ALL cell lines, Jurkat and KOPT-K1 harbouring activating Notch1 mutations, displayed a differential outcome. In Jurkat cells, HH stimulation resulted in activating Notch1 and decreasing MYC expression as well as in suppressing the formation of cell colonies, while HH stimulation was ineffective in KOPT-K1 cells [61]. That observation emphasizes the cell context dependency of these signals and at the same time likely discloses a novel relationship between the pathways of HH and Notch in T-ALL.

In T-ALL, Notch1 mutations frequently require persistent Notch signaling to promote growth and survival of leukemic cells. New gene products that enhance signaling of leukemia-associated Notch1 mutants have been also identified [62]. In murine T-cell leukemia models, MafB transcription factor enhances leukemogenesis of naturally occurring Notch1 mutants, by decreasing disease latency and/or increasing penetrance [62]. Moreover, Notch signaling may contribute to chemotherapy resistance [63] in T-ALL, thus suggesting a strategy of Notch inhibition as a targeted therapy in disease progression. Given the important role of Notch signaling in T-ALL, a number of novel Notch inhibitory strategies have been undertaken [64, 65]. Resistance mechanisms are often driven by oncogenic pathways that crosstalk with Notch signals. In this regard, PTEN loss can promote PI3K/mTOR signaling [66, 67], or FBWX7 loss can augment Notch1 signaling as well as the stabilization and protein level of the transcription factor MYC [20, 47]. Finally, the drug resistance to Notch1 inhibitors can also be ascribed to metabolic reprogramming in T-ALL. In that context, inhibition of Notch1 signaling induces a prominent inhibition of glutaminolysis and triggers autophagy as a salvage pathway to support T-ALL metabolism [67].

The role of hyperactive Notch signals is a milestone in T-ALL pathogenesis, but still remains elusive. The continuous crosstalk of the Notch ligand/receptor complex with other pathways sustains BM infiltration and drug-resistance and should be further intensively researched.

## 5. The SDF-1/CXCR4 Axis in the Pathogenesis of B-ALL

The SDF-1/CXCR4 axis plays an important role in normal B-cell lymphopoiesis mainly during cell migration and homing of hematopoietic stem cells into the BM niches [68–70]. CXCR7 receptor has been identified as the second receptor for SDF-1. This atypical chemokine receptor binds SDF-1 with higher affinity and probably tunes the responses after SDF-1 binding to CXCR4 [71].

This axis is also involved in many different types of cancer, including hematological malignancies [13] where it seems to have a role in the propagation of leukemic cells [72] and in their interaction with the components of a tumor microenvironment. Leukemic cells and leukemic stem cells actively interact with SDF-1-expressing organs including the BM, liver, thymus, lymph nodes, and brain. The characterization of BM niches in ALL leukemia is essential to fully understand ALL pathogenesis, which still remains obscure and needs to be studied further. As other hematological malignancies, B-ALL results from a rare population of cancer stem cells, defined also as leukemia-initiating cells, which are carrying the first oncogenic mutations.

Various studies have suggested a role to the SDF-1/CXCR4 axis in the etiology of ALL, but only few correlated it with the pathogenesis of B-ALL [73] (Table 2). Moreover, although CXCR7 influences the migration of B-cells during maturation [74] the role it plays in B-ALL is still unknown.

Currently, studies in patients support the presence of high CXCR4 expression on the cell surface of leukemic blasts and BM infiltration [75–78] in B-ALL. Some of these experimental findings associate high levels of CXCR4 with an increased incidence of relapse and poor outcome (shorter disease-free survival) [77–80]. Relapse of B-ALL occurs when leukemic B-cell precursors egress from the BM microenvironment, reach the blood circulation, and infiltrate extramedullary organs. CXCR4 overexpression has also been demonstrated in lymphoblasts from patients with infiltration of leukemic cells into the spleen, liver, lymph nodes, CNS, testicles, and skin [81–84]. In B-CLL, CXCR4 expression is a direct transcriptional target of Notch1 [28]. Studies by Alsadeq et al. [83] showed that Zap70 can control the expression of CXCR4 and CCR7 and the high expression levels of these chemokine receptors are correlated with CNS infiltration during B-ALL relapse. In contrast, the experimental findings of Williams et al. [84] did not reveal any relation between chemokine receptor levels (investigating also CXCR7 levels) and infiltration into the CNS of xenotransplanted primary B-ALL samples. In their study, although the inhibition of the SDF-1/CXCR4 axis caused lower liver and BM infiltration, no differences were observed regarding the CNS infiltration. For this discrepancy, the authors hypothesize that just different populations of leukemic cells may have this capacity.

Given the implication of the axis in cell migration and homing of leukemic cells, CXCR4 has become a therapeutic target and CXCR4 antagonists in B-ALL were the object of studies by different groups. In mice, a rapid mobilization of leukemic cells to peripheral blood and a significant reduction of precursors B-ALL cells was observed by blocking the SDF-1/CXCR4 axis [85]. The study of Randhawa et al. [86] showed that CXCR4 inhibition could increase sensitivity to chemotherapy in B-ALL. Their results suggest that the disruption of the axis SDF-1/CXCR4 could sensitize leukemic cells normally protected in the BM microenvironment to the cytotoxicity of standard chemotherapy. Finally, they reported that B-ALL cells are negative for CXCR7 protein expression, arguing against a role of CXCR7 in B-ALL. Overall, this data suggests the supportive role of BM stroma in B-ALL cell maintenance.

Thus far, reports on SDF-1 serum levels in leukemic patients are few. The study of Mowafi et al. [87] evaluated SDF-1 levels in serum from ALL children and reported that SDF-1 may have a role in leukemic cell proliferation and survival during childhood pre-B-ALL. On the other hand, Khandany et al. [88] evaluated SDF-1 serum levels in adult ALL patients prior to and post BM transplantation (BMT). They showed that the serum levels of SDF-1 were significantly increased in ALL patients compared to the controls, while after BMT in ALL patients SDF-1 levels were decreased, suggesting that SDF-1 is important for the pathogenesis of ALL and might be used as a pivotal biological marker in the diagnosis of leukemia. The most recent report of SDF-1 serum levels is by the studies of van den Berk et al. [80] performed in precursor B-cell ALL patients. The authors found that SDF-1 serum levels were low at the time of diagnosis, when leukemic cells were numerous in the BM, and increased after chemotherapy (up to levels seen in nonleukemic controls). These evidences suggest that leukemic cells can modulate SDF-1 release. In concomitance with low SDF-1 serum levels, they reported elevated G-CSF levels, a factor known to mobilize HSC and myeloid cells from the BM into the blood compartment by downregulating CXCR4 expression and attenuating their responsiveness to SDF-1 [89]. Those two findings may sustain the hypothesis of how leukemic cells migrate from the BM into the blood and contribute to metastasis.

## 6. The SDF-1/CXCR4 Axis in the Pathogenesis of T-ALL

The activation of the SDF-1/CXCR4 axis is a critical event for the migration and retention of leukemia cells within BM, for extramedullary colonization and for the maintenance of minimal residual disease (MRD), as demonstrated in acute myeloid leukemia [90]. High surface CXCR4 have been observed on all T-ALL subtypes, also comprising high-risk early T precursors ALL (ETP-ALL) [16]. To date, only recent reports highlighted the importance of Notch and CXCR4 signalings in T-ALL propagation and invasion (Table 2). Recently, interesting findings demonstrated a strong link between Notch1-induced T-ALL progression and enhanced CXCR4-dependent infiltration in the BM [15, 16]. In this leukemic microenvironment, the release of SDF-1 by BM stroma creates a vascular endothelial niche which is perfect for the maintenance of T-ALL cells [16], possibly by enhancing the adhesion ability of tumor cells. Moreover, CXCR4 play a major role in leukemia initiating activity and T-ALL propagation in vivo, by modulating not only cell motility but also survival and proliferation in T-ALL cells. Additionally, the study of Passaro et al. [15] demonstrated that enhanced CXCR4 cell surface expression depends upon calcineurin-dependent expression of cortactin, an actin-binding protein involved in cytoskeleton dynamic processes [15]. Evidences reported by our group, in a Notch3-induced T-ALL model, showed the anomalous presence of Notch3^+^CXCR4^+^CD4^+^CD8^+^in the BM and spleen. These cells have been abnormally found in blood circulation and showed the ability to rapidly infiltrate the BM of immunocompromised mice [17, 54]. The enhanced CXCR4 surface expression was essentially associated to mechanisms regulating the trafficking of the receptor in a plasma membrane [15, 54]. In all papers [15–17], it was not hypothesized that CXCR4 gene expression could be transcriptionally regulated by Notch, although it cannot be excluded in a specific T-cell context. In contrast, transcriptional activation of CXCR7 gene by Notch1-RBPjK complexes has been described in T-ALL [91]. Interestingly, the study of Wang et al. [91] individuated *cxcr7* within the group of genes with high-dynamic regulatory potential. Additionally, high levels of CXCR7 transcripts were found in malignant ALL cells and cell lines and in the T-ALL subtypes [63, 92, 93],, although little surface expression of CXCR7 was detected in mouse Notch1-induced T-ALL cells with high surface levels of CXCR4 [15]. Given the importance of CXCR4 in T-ALL propagation and the ability of CXCR7 as a scavenger receptor to modulate CXCL12 in the extracellular milieu, it will be of great interest to deepen our knowledge in that receptor/ligand interplay. An additional layer of complexity of this receptor system could be the hyperactive noncanonical HH signaling, which negatively regulates CXCL12 and Jagged1 in the context of myeloproliferative neoplasms [94].

All papers so far evidenced that targeting CXCR4 in Notch-induced T-ALL reduces tumor growth and BM infiltration in the animal models [15, 16, 54], suggesting the central role of CXCR4 in Notch-triggered T-cell leukemia progression. The propensity of leukemic cells to invade the CNS is associated to a bad prognosis in T-ALL. In fact, CNS-directed therapy reduced the frequency of disease recurrence [95]. Interestingly, it has been demonstrated that CXCR4 inhibition, by using pharmacological antagonism (AMD3100) or Notch1IC-transduced and CXCR4-deficient hematopoietic progenitors, dramatically impacts BM engraftment of T-ALL cells [96]. Similarly, AMD3100 administration can prevent preleukemic BM infiltration in a Notch3-induced T-cell leukemia model [17] or AMD3465 in Notch1-induced T-ALL. Therefore, even as single drugs, they have a significant antileukemia activity. Additionally, small molecules blocking the CXCR4/SDF-1 axis have been developed and may be proposed as an alternative strategy to overcome BM-induced chemoresistance in acute leukemia [97].

Therefore, CXCR4 antagonism can be hypothesized in T-ALL treatment, also against early circulating leukemia cells. To date, combined therapy with CXCR4 antagonist (BL8040) and nelarabine as a salvage therapy for patients with relapsed/refractory T-ALL/LBL is currently open (NCT 02763384) [13].

## 7. Notch and CXCR4 Hand-In-Hand toward ALL Progression

Leukemic ALL precursors extravasate in order to gain access to extramedullary organs frequently exploiting inflammatory adhesion molecules. In T- and B-ALL progression, extravasation represents a critical event still poorly dissected. Multiple chemokines participate to ALL precursor infiltration, possibly driving to tissue-specific metastasis [54]. T-ALL infiltration mechanisms envisage SDF-1, CCL19 (CNS, lymph nodes), CCL25 (small bowel), CCL17/22, and CCL27/28 (skin) chemokines. CXCR4 may be a unifying target in B- and T-ALL infiltration, since high CXCR4 expression is found in B- and T-ALL cells and is correlated with higher incidence of relapse [14] (Table 2). CXCR4 hyperexpression, enhanced by a direct transcriptional effect of Notch1 [28, 98], is associated with B-cell infiltration into the spleen, liver, lymph nodes, CNS, and testicles [80] [84]. Similarly, CXCR4 hyperexpression mediates T-ALL infiltration in the BM, spleen, liver, lung, and CNS as the preferred site[14] (Table 2). CXCR4 and CCR7 are the only accredited chemokine receptors associated to leukemic CNS infiltration in Notch-induced T-ALL [96, 99] and B-ALL [83]. Although still debated, chemokine receptor CCR7 together with CCR6 seems to be not required for meningeal infiltration by Notch1-induced T-ALL [96, 99]. In keeping with recent findings, the authors suggest the possible relevance of CXCR4 as a pharmacological target for T-ALL therapy, further indicating the importance of CXCR4, but not of CCR6 and CCR7, in CNS invasion. Therefore, inhibition of CXCR4 activity could prevent the devastating effect in CNS.

The SDF-1/CXCR4 axis has a prominent role in lymphostromal interactions occurring in the thymus and in the BM of Notch-induced T-ALL [16]. In that niche, SDF-1 constitutively produced by vascular endothelial cells sustains T-ALL maintenance. The SDF-1/CXCR4 pathway also modulates regulatory T-cells (Treg) trafficking from the BM. Of note, NF-*κ*B transcription factor mediates the enhanced generation of Treg in a Notch3-dependent T-ALL mouse model, thus suggesting a Notch3/CXCR4 synergism that facilitates tumor development by suppressing antitumor immune response [54, 100]. It is believed that B- and T-ALL cells promote *ΒΜ* remodeling. In B-ALL, expression of SDF-1 in BM lymphoid niches under proinflammatory settings is reduced to allow the dominance of malignant cells over normal differentiation [101]. In T-ALL, stromal Notch activation negatively regulates SDF-1 within the stem and lymphoid niches promoting a Notch-dependent malignant progression [14, 102]. In addition, the high level of HH ligands released by T-ALL cells, which promotes their growth and stimulates DLL4 expression, and the coexpression of HH receptor and SDF-1 in stromal cells of the *ΒΜ* could all contribute to maintain leukemic cells in the tumor niche.

In T-ALL, Notch1 can induce metabolic reprogramming, a mechanism involved in resistance to anti-Notch therapies [67]. Inhibition of Notch signaling in T-ALL induces a metabolic shutdown, with inhibition of glutaminolysis and triggering of autophagy, a salvage pathway supporting leukemia cell metabolism. Interestingly, CXCR4-mediated signaling induces autophagy and influences AML cell survival and drug resistance [103]. Overall, these findings can suggest that Notch and CXCR4 could converge on metabolic pathways to further sustain leukemia progression but also unveil a new therapeutic target that may be common to B- and T-ALL. Further studies are required.

## 8. Conclusion and Perspectives

The pathways of Notch and CXCR4 are emerging as potential therapeutic targets in an expanding range of T and B lymphoproliferative disorders. Mutational and nonmutational mechanisms of Notch activation or ligand dependence by leukemia cells/stroma interactions should be considered. Future research should identify the role played by Notch ligands in driving the growth and expansion of lymphoid malignancies in specific niches. Primary therapy can select for Notch-resistant or Notch-independent tumors, seen in the relapsed/refractory setting. Therefore, CXCR4 may represent a second hit to tackle ALL. For that reason, we need to know the molecular mechanisms accounting for receptor phosphorylation, ubiquitination, recycling, and internalization rate, as defects in endocytic trafficking of CXCR4 may contribute to cancer progression.

Several clinical trials (Phase I/II), mainly in AML patients, proposed that the addition of CXCR4 inhibitors to current therapies has a clinical benefit [13].

Both Notch and CXCR4 receptor signaling are involved in ALL chemoresistance. Therefore, combination therapies with the aim at impacting these pathways may have a stronger therapeutic effect. A deeper and more nuanced understanding of the crosstalk of these pathogenic signals in ALL development will provide new findings to be translated into clinical application.

## Figures and Tables

**Table 1:** A comparison between different mechanisms deregulating the Notch receptors in B-cell acute lymphoblastic leukemia (B-ALL) and in T-cell lymphoblastic leukemia (T-ALL).

Genes	B-ALL	T-ALL
NOTCH1	No clear evidence of mutations Upregulated [41]	Frequent activating mutations (60% of patients) [8, 44]

NOTCH2	No clear evidence of mutations Upregulated in BM-derived precursor-B ALL cells [104]	Activating mutations only in adults [45]

NOTCH3	No clear evidence of mutations Upregulated [40–42] Epigenetic modifications: hypermethylated [36]	Rare activating mutations [9] Frequent hyperexpression [37, 46] Epigenetic modifications: hypomethylated or unmethylated [37]

NOTCH4	No clear evidence of mutations Upregulated [40, 41]	No clear evidence of mutations or hyperexpression

BM: bone marrow.

**Table 2:** High CXCR4 cell surface expression is a common hallmark in B-cell acute lymphoblastic leukemia (B-ALL) and in T-cell lymphoblastic leukemia (T-ALL) propagation and disease progression.

High expression of CXCR4 in B-ALL is correlated to the following:	High expression of CXCR4 in T-ALL is correlated to the following:
BM infiltration [75–78]	BM infiltration in combination with Notch1 or Notch3 hyperexpression [15–17, 96]
Liver, spleen, lymph nodes, testicles, and skin infiltration [81–84]	Liver, spleen, and lung infiltration [37, 96, 99]
CNS infiltration in combination with CCR7 [83]	CNS infiltration in Notch1-induced T-ALL [96]
Stroma-mediated drug resistance [86]	Stroma-mediated drug resistance and maintenance of the disease [13, 16, 46]
Higher incidence of relapse and poor outcome (shorter disease-free survival) [77–80]	Higher incidence of relapse [14, 15]
No clear evidence of CXCR7 cell surface expression [86]	Low cell surface expression of CXCR7 in Notch1-induced T-ALL [16]

BM: bone marrow; CNS: central nervous system.
